# Prevalence and Associated Factors of Overweight/Obesity Among Women in the Reproductive Age Group (15–49) in Gondar Town, Northwest Ethiopia, 2022: A Cross‐Sectional Study

**DOI:** 10.1002/hsr2.70810

**Published:** 2025-05-06

**Authors:** Mequanente Dagnaw, Melkie Mekonnen, Esmeal Ali Muhammad, Solomon Mekonnen Abebe

**Affiliations:** ^1^ Department of Epidemiology and Biostatistics, Institute of Public Health University of Gondar Gondar Ethiopia; ^2^ Department of Medical Biotechnology, Institute of Biotechnology University of Gondar Gondar Ethiopia; ^3^ Department of Human Nutrition, Institute of Public Health University of Gondar Gondar Ethiopia

**Keywords:** body mass index, Ethiopia, overweight/obesity, reproductive age women

## Abstract

**Background and Aims:**

Overweight/obesity is increasing at an alarming rate throughout the world and has now become a global epidemic. According to a recent research report, the burden of obesity is increasing in developing countries; however, no significant reduction has been seen in developed countries over the past few decades. Furthermore, there is limited information about overweight/obesity. The main objective of this study was to assess the prevalence and associated factors of overweight/obesity among reproductive age women.

**Methods:**

A total of 866 reproductive women participated in the study. The collected data were coded and entered into Epi‐Data version 4.6.0 and exported to STATA version 17 software packages for analysis. Binary logistic regression was used to identify factors associated with overweight/obesity among reproductive age women. Variables with a *p*‐value < 0.2 in the bivariate analysis was further fitted into multivariate analysis for controlling the possible effect of confounders, and finally the variables that were a *p*‐value of < 0.05 on the bases of odd ratio (OR) with 95% confidence interval (CI) was considered significantly associated with overweight/obesity and identified based on odds ratio (OR), with 95% confidence interval (CI) and *p*‐value (*p* < 0.05).

**Result:**

The study's response rate was 96% and the overall prevalence of overweight/obesity was 21% [95% CI: 18.00, 24.00]. Being parity [AOR = 3.75, 95% CI: 2.06, 6.81], parity (≥ 2) [AOR = 8.16, 95% CI: 4.63, 14.37], late age at menarche (≥ 14 years) [AOR = 1.65, 95% CI: 1.13, 2.41], and ever used family planning [AOR = 1.50, 95% CI: 1.01, 2.23] were factors significantly associated overweight/obesity.

**Conclusion:**

In this study, overweight/obesity was high. Late age of menarche, parity, and ever using family planning were factors affecting overweight/obesity in reproductive age women. Healthcare workers should raise awareness among reproductive‐age women about the impacts of overweight/obesity.

## 1 Introduction

Overweight/obesity has become a major public health problem both in developed and developing countries [1, 2]. Overweight and obesity are the fifth leading risk factors for global deaths. At a minimum point, 3.4 million adults die each year as a result of being overweight or obese [3]. Overweight and obesity provide substantial difficulties in both high and low‐to‐middle‐income nations [4]. These disorders have a severe and destructive effect on women, impacting their health, as well as the health of their children [5]. Pregnant women who have a higher body weight or are obese are at a greater risk of experiencing issues during pregnancy. These complications include gestational diabetes, high blood pressure, pre‐eclampsia, excessive bleeding after childbirth, the need for assisted delivery, infections at the surgery site, having a baby with low birth weight, and the occurrence of congenital disabilities are becoming a result of overweight/obesity. In addition, the diminished quality of life caused by being overweight or obese places more medical, psychological, and social burdens on society [6].

Excess weight and obesity are linked to severe medical diseases such as high blood pressure, abnormal levels of fats in the blood, heart disease, stroke, diabetes, gallbladder disease, joint inflammation, and some types of cancer [7]. It is worth mentioning that women are more likely to be affected by several health disorders associated with obesity, including osteoarthritis, breast and endometrial malignancies, cardiovascular and gallbladder diseases, infertility, and gynecological complications, as well as experiencing stigma and discrimination. Research indicates that the global occurrence of overweight and obesity in children and adolescents is estimated to be 13.5% [8]. In Africa, the issue of undernutrition is still a major concern. However, there has been a growth in the number of people who are overweight or obese, with a prevalence of 8.5% in 2010. This number is expected to increase to 12.7% by 2020 [9, 10].

Obesity and overweight rates are increasing in Ethiopia, and the government has acknowledged it as a rapidly growing public health issue that is not caused by infectious diseases [11, 12]. Nevertheless, there is a dearth of studies conducted in the Amhara region. The examination of data from the Ethiopia Demographic and Health Survey (EDHS) indicated that the occurrence of overweight and obesity among adult women in Addis Ababa is high. A separate study in Addis Ababa demonstrated a significant 28% rise in overweight and obesity [13].

Previous research issues involve the utilization of diverse data sets with different levels of representativeness and techniques, encompassing socio‐demographic information, levels of physical activity, nutritional components, reproductive health factors, skinfold thickness, and BMI measurements are the most risk factors. It is essential to tackle these problems to adequately assess the incidence of overweight and obesity, especially among women of reproductive age.

To address non‐communicable diseases (NCDs) such as overweight and obesity, the World Health Organization (WHO) suggests implementing multi‐sectoral public policies that encourage the creation of environments that promote health, as well as focusing on health education, health literacy, and tobacco control [14]. Nevertheless, there are still other factors that impact the occurrence of overweight and obesity in women of reproductive age, which highlights the need for comprehensive approaches to prevent and manage these conditions.

In Ethiopia, there is still food insecurity, diet, and weight outcomes problems, as well as the effectiveness of intervention strategies. The findings of this study can provide significant empirical backing for program designers, politicians, researchers, and organizations engaged in the prevention of non‐communicable diseases linked to excessive weight and obesity. The purpose of this study is to assess the prevalence and associated factors of overweight/obesity among women in the reproductive age group (15–49) in Gondar town.

## 2 Methods

### 2.1 Study Design and Period

A community‐based cross‐sectional study was conducted to determine the prevalence and associated factors of overweight/obesity among reproductive age women in Gondar Town, Northwest Ethiopia, from January 2022 to July 2022.

### 2.2 Study Area

The study was conducted in Gondar town. The town has a total population of 432,191(101,910) and it has total six Sub cities, such as Markie 68,229 (16,088), Jan Tkele 175,221 (41,317), Fasil 68,432 (16,136), Arada 68,418 (16,132), Azezo 110,367 (26,024), Zobele 73,798 (17,401) with total populations and reproductive women population amount. Gondar city has 24 public health facilities (3 hospitals, 8 HCs, and 14 health posts) to offer healthcare services. A total of 306 HWs with different professional disciplines are working in those healthcare facilities.

### 2.3 Population

#### 2.3.1 Source Population

All women of reproductive age living in Gondar town.

#### 2.3.2 Study Population

All women of reproductive age living in the selected sub‐city of Gondar town.

### 2.4 Inclusion and Exclusion Criteria

#### 2.4.1 Inclusion Criteria

The present study focused mainly on all women aged 15–49 years included in the study.

#### 2.4.2 Exclusion Criteria

The pregnant women, having deformed (spinal anomalies) reproductive organs and those who had given birth within 6 months before the data collection, were excluded from this study.

### 2.5 Sample Size and Sampling Procedures

The study's sample size was determined using the formula for estimating a single proportion, assuming a 95% confidence interval (*z*), a 4% marginal error (*d*), and a proportion of 20.6% from the previous study [13].

Based on this assumption, the sample size is determined to be 393. After accounting for the 10% nonresponse rate, the total sample size is 433. Proportional allocation was employed to ensure that the six sub‐cities, which were selected randomly, maintained proportionality. Next, study participants were chosen from two sub‐cities, with a design effect of two. The ultimate sample size was determined to be 866, obtained by multiplying 433 by 2. The study region consists of a diverse sample population from various Kebles. A multi‐stage random sampling technique was used to select study participants. A total of 866 study participants were selected from the two sub‐cities using a simple random sampling technique. From these, 272 samples were taken from Maraki sub‐city and 594 were from Jantkel sub‐city. Then, 272 and 594 samples of Maraki and Jantkel sub‐city were distributed to the respective Kebles of the sub‐city in both selected sub‐cities. The number of participants was allocated proportionally based on the population at each sub‐city.

### 2.6 Study Variables

Dependent variable: Overweight/obesity (Yes/No)

Independent variable:

Socio‐demographic characteristics: Age, education status, family size, marital status, ethnicity, religion, occupational status, and wealth index.

Behavioral factors: Cigarette smoking, alcoholic drink, dietary habit, physical activity, dietary diversity, contraceptive use, family history of overweight or obesity, and means of transportation.

### 2.7 Operational Definition

Overweight: A BMI‐for‐age greater than + 1 standard deviation (SD) above the median of the WHO Growth Reference for this age group is approximately equivalent to a BMI of 23 kg/m² but less than 26 kg/m² at 15 years, which corresponds to the adult threshold for overweight [15, 16].

Obesity: Obesity is defined as a BMI‐for‐age greater than + 2 standard deviations above the WHO Growth reference median for children and adolescents aged 5–19 years. For a 15‐year‐old female, this corresponds to a BMI above 28.2 kg/m², indicating obesity [15, 16].

Dietary practice: Dietary diversity was usually measured by summing the number of foods or food groups consumed over a reference period. The reference period usually ranges from 1 to 3 days, but 7 days is also often used, and periods of up to 15 days have been reported [17].

Level of physical activity: It is the measurement value of self‐report questionnaires, self‐report activity diaries/logs, and direct observation [18].

Sedentary behaviors: Sedentary behavior was often assessed by the amount of time adults spend viewing TV or other technologically based sedentary behaviors, such as computer use or playing video games. However, these sedentary behaviors provide only a partial picture of overall levels of sedentary behavior in a typical waking day [19].

Wealth status: The household asset was divided into Tertiles (lowest, middle, and richest) [20]

Substance use: Substance use as a behavior can be described by measuring three parameters: (1) quantity, (2) frequency, and (3) duration of quantity was a measure of dose and was represented by the amount per occasion. Quantity was also a measure of the style of use [21].

### 2.8 Data Collection Procedures

The data was gathered by a structured interviewer‐administered questionnaire obtained from a review of previous studies with comparable objectives. The questionnaire was then adapted from the World Health Organization (WHO) to make it acceptable for use in the specific study area. The survey encompassed socio‐demographic parameters, behavioral factors, physical activity, nutritional choices, and women's reproductive aspects, which were considered independent variables. Eight BSc nurses and two supervisors were enlisted to gather data. The data collectors and supervisors received 2 days of training on the study's purpose and how to approach the study participants and conduct anthropometric measurements.

#### 2.8.1 Anthropometric Measurements

Height was measured using a portable stadiometer. Participants were barefoot, with their legs straight, shoulders relaxed, and eyes looking straight ahead in the horizontal plane. Before measurement, they were instructed to inhale deeply, hold their breath, and maintain an erect posture. Height was recorded to the nearest 0.1 cm. For individuals with kyphosis, where height measurement was challenging, arm span was used as an alternative. Arm span was measured as the distance from the middle fingertip of the left hand to that of the right hand when both arms were fully extended horizontally. This measurement was then converted to a height equivalent [22]. Weight was measured using a portable digital scale, with participants wearing minimal clothing and standing at the center of the scale's platform. Weight was recorded to the nearest 0.1 kg, with each measurement taken three times and then averaged. Body Mass Index (BMI) was calculated by dividing body weight (kg) by height (m²). In this study, nutritional status was classified as normal for a BMI between 18.5 kg/m² and 24.9 kg/m², while individuals with a BMI below 18.5 kg/m² were considered malnourished [23].

Mid‐upper arm circumference (MUAC) was measured using a non‐stretch adult MUAC tape. The participant's left arm was first flexed at a 90‐degree angle at the elbow, and the midpoint between the lateral acromion and the distal olecranon was identified and marked. The arm was then relaxed, and the MUAC tape was wrapped around the marked midpoint. Measurements were recorded to the nearest 0.1 cm. A MUAC of less than 21 cm was classified as malnourished [24].

### 2.9 Data Quality Control

The questionnaires were pre‐tested a week before the actual data collection days for 10% of the sample size on the same sampled unit of the study, but in a sub‐city which was not selected for the study. And modification was done accordingly. Data collectors and supervisors were trained by the principal investigator. During data collection, trained supervisors were checked in the field to see how the data collectors were doing their tasks. At the end of each data collection day, the principal investigator also checks the completeness of filled questionnaires. In case a questionnaire was found to be missing some information, the respective data collectors were to go back to the respondents concerned to complete the questionnaire.

### 2.10 Data Management and Statistical Analysis

Statistical analyses were performed using STATA version 17. Socio‐demographic characteristics and behavioral factors were described in weighted frequency and percentage. Bivariate analysis using *χ* ^2^‐tests was used to assess associations between independent variables (socio‐demographic characteristics and behavioral factors) and overweight/obese. Variables associated with overweight and obesity at *p*‐value 0.10 or that had a potential confounder variable (e.g., women's age, household wealth index, alcohol consumption, and place of residence) were included in the final multiple logistic regression analyses. Simple logistic regression was used to determine the magnitude of the effect of associations between overweight and obesity with socio‐demographic characteristics and behavioral factors. Results are reported as odds ratios (OR) with 95% confidence intervals (CI). Binary logistic regression was then used to assess independent factors associated with overweight and obesity after adjusting for other potential confounding factors in the model. Results from the final adjusted model are reported as adjusted odds ratios (AOR) with 95% CI and corresponding *p*‐values. Multicollinearity between independent variables was checked before fitting the final regression model, including women's age, number of children ever born, education, household wealth index, occupation, marital status, place of residence, and geographical regions. The result of the evaluation of variance inflation factor (VIF) scores after fitting an Ordinary Least Squares regression model with the mean = 1.44 indicated no collinearity concerns.

### 2.11 Ethical Consideration

The study was approved and given its ethical clearance by the Institutional Ethical Review Committee Board of the University of Gondar (Reference No/IPH 24/07/2022). Informed consent was not used because the study was a retrospective study of secondary data of the PLHIV. But by keeping the information that was extracted private and making sure that it could only be utilized for study‐related objectives, the data's secrecy was preserved. The participant's informed consent waiver was authorized by the institutional ethical review board of the University of Gondar. Additionally, all procedures were carried out in accordance with the necessary rules and laws.

## 3 Result

### 3.1 Socio‐Demographic Characteristics of Study Participants

The response rate of the study was 96%. The final analysis comprised 826 women in the reproductive age category, with a response rate of 100%. The age group of 15–18 comprised nearly one‐third of the study participants, specifically 268 (32.45%). 391 (47.34%) of the sample were married, less than half of the study participants. Regarding educational attainment, 358 patients (43.34%) had completed secondary education. The occupational status of approximately 265 individuals (32.08%) was job‐sick (Table 1).

**Table 1:** Sociodemographic characteristics/variables of prevalence and associated factors of overweight/obesity among women in the reproductive age group (15–49) in Gondar Town, 2022 (*N* = 826).

Variables	Category	Frequency (*N*)	Percentage (%)
Age	15–18	268	32.45
	19–30	193	23.37
	31–40	272	32.93
	41–49	93	11.26
Ethnicity	Amhara	759	91.89
	Tigray	27	3.27
	Others	40	4.84
Marital status	Single	291	35.23
	Married	391	47.34
	Divorced	102	12.35
	Widowed	42	5.08
Religion	Orthodox	710	85.96
	Muslim	97	11.74
	Protestant	19	2.30
Family size	1–3	479	59.99
	4–6	317	38.38
	7–8	30	3.63
Family type	Nuclear	226	27.36
	Joint/Extended	600	72.64
Educational status	Unable to read and write	116	14.04
	Primary education	202	24.46
	Secondary education	358	43.34
	Higher education and above	150	18.16
Occupation	Student	265	32.08
	Housewife	126	15.25
	Private employee	154	18.64
	Governmental employee	93	11.26
	Merchant	57	6.90
	Daily laborer	62	7.51
	Student	69	8.35
Wealth index	Poor	280	33.9
	Medium	252	30.5
	Rich	294	35.6

### 3.2 Wealth Index Characteristics of Study Participants

Most study participants, specifically 570 individuals (69%), had no private residence. 813 (98%) participants were roofed with corrugated iron sheets. Most study participants, specifically 498 individuals (60.29% of the overall sample), possessed stoves (Table 2).

**Table 2:** Wealth index characteristics/variables of prevalence and associated factors of overweight/obesity among women in the reproductive age group (15–49) in Gondar Town, 2022 (*N* = 826).

Variables	Category	Frequency (*N*)	Percentage (%)
Private house	Yes	256	30.99
	No	570	69.01
Roof	Corrugated iron sheet	813	98.43
	Thatch	13	1.57
Wall	Mud	676	81.84
	Cement	144	17.43
	Bricks	6	0.73
Floor	Soil	422	51.09
	Cement	351	42.49
	Ceramic	53	6.42
Electricity	Yes	256	30.99
	No	570	69.01
Radio	Yes	813	98.43
	No	13	1.57
Television	Yes	676	81.84
	No	144	17.43
Mobile	Yes	721	87.29
	No	105	12.71
Nonmobile telephone	Yes	26	3.15
	No	800	96.85
Refrigerator	Yes	208	25.18
	No	618	74.82
Chair	Yes	652	78.93
	No	174	21.07
Table	Yes	721	87.29
	No	105	12.71
Bed	Yes	800	96.85
	No	26	3.15
Stove	Yes	498	60.29
	No	328	39.71

### 3.3 Food and Dietary‐Related Characteristics of Study Participants

Of the participants, 348 individuals consumed their food weekly, which accounts for 42% of the total. Out of the overall sample, 377 people (46%) who consumed veggies once each month were married (Table 3).

**Table 3:** Food and dietary‐related characteristics/variables of prevalence and associated factors of overweight/obesity among women in the reproductive age group (15–49) in Gondar Town, 2022 (*N* = 826).

Variables	Category	Frequency (*N*)	Percentage (%)
How many times do you eat	Twice a week	58	7.02
	Three times	736	89.10
	Four times	32	3.87
Do you drink soft drinks	Yes	404	48.31
	No	422	51.69
How frequent	One time	242	29.30
	Two times	115	13.92
	Three times	47	5.69
	None	422	51.09
Frequently citrus fruit	Once a week	109	13.20
	Twice a week	41	4.96
	More than twice per week	85	10.29
	Once per month	377	45.64
	Never	214	25.91
Dietary diversity	< 5 food groups	718	86.9
	≥ 5 food groups	108	13.1

### 3.4 Anthropometric Measurements of Characteristics of Study Participants

The final study included 826 women in the reproductive age group. Six hundred thirty‐six research participants, accounting for 77% of the responses, had a normal BMI (Table 4).

**Table 4:** Anthropometric measurements characteristics/variables of prevalence and associated factors of overweight/obesity among women in the reproductive age group (15–49) in Gondar Town, 2022 (*N* = 826).

Variables	Category	Frequency (*N*)	Percentage (%)
Weight (kg)	40–50	243	29.42
	51–60	383	46.37
	61–70	149	18.04
	71–79	51	6.17
Height (m)	1.40–1.50	131	15.86
	1.51–1.60	492	59.56
	1.61–1.70	203	24.58
BMI	Underweight (< 18.5 kg/m²)	18	2.18
	Normal weight (18.5–24.9 kg/m²)	636	77
	Overweight (23 and 26 kg/m²)	160	19.37
	Obesity (> 28 kg/m²)	12	1.45

### 3.5 Prevalence of Overweight/Obesity Among Women in Study Participants

The prevalence of overweight/obesity among reproductive age was 172 (20.8%) with [95% CI: 18.00, 24.00]. About 654 (79.2%) of reproductive‐age mothers had underweight and normal weight.

### 3.6 Factors Associated With Overweight/Obesity

In the bivariate analysis, the factors that showed a significant association with overweight/obesity with a *p*‐value of ≤ 0.2 were parity, age of menarche, and ever‐used family planning. These factors were then included in the multivariate analysis. The multivariate analysis identified women with a parity (para one) of 3.75 times [AOR = 3.75, 95% CI: 2.06, 6.81] as the relevant variables. Nulliparous women have a greater likelihood of becoming overweight or obese compared to women with a parity of 2 or more. The risks of developing overweight or obesity among women with a parity of 2 or more were 8.16 times higher [AOR = 8.16, 95% CI: 4.63, 14.37]. Compared to women with no children, women who reached menarche at the age of 14 or older had a 1.65 times higher likelihood of developing overweight or obesity. This was determined with an adjusted odds ratio (AOR) of 1.65 and a 95% confidence interval (CI) of 1.13 to 2.41. Women with an age of menarche less than 14 years had a higher risk compared to women whose age of menarche was higher. Additionally, women who have ever used family planning had a 1.50 times higher risk [AOR = 1.50, 95% CI: 1.01, 2.23]. The multivariate logistic regression analysis revealed a significant association between the use of family planning and a higher likelihood of acquiring overweight or obesity in women than those who had never used family planning (Table 5).

**Table 5:** Both bivariate and multivariate results of factors affecting overweight/obesity among women of reproductive age in Gondar Town, North West Ethiopia, 2022 (*N* = 826).

Variables	Categories	Overweight/Obesity		COR (95% CI)	AOR (95% CI)	*p* value
		Yes	No			
Marital status	Single	59	232	0.64 (0.31, 1.32)	0.79 (0.35, 1.82)	0.582
	Married	83	308	0.67 (0.33, 1.373)	0.84 (0.38, 183)	0.657
	Divorced	18	82	0.54 (0.23, 1.242)	0.76 (0.30, 1.89)	0.549
	Widowed	12	30	1	1	
Occupation	Job sicker	58	207	1.21 (0.62, 2.34)	1.15 (0.56, 2.36)	0.701
	Housewife	20	106	0.81 (0.38, 1.76)	0.67 (0.29, 1.51)	0.328
	Private	33	121	1.18 (0.57, 2.40)	1.08 (0.50, 2.32)	0.852
	Governmental	16	77	0.90 (0.40, 2.01)	0.82 (0.34, 1.94)	0.644
	Merchant	19	38	2.15 (0.95, 4.88)	1.82 (0.75, 4.43)	0.186
	Daily laborer	13	49	1.14 (0.49, 2.69)	1.12 (0.45, 2.81)	0.807
	Student	13	56	1	1	
Parity	Null parity	18	299	1	1	
	Parity	55	192	4.76 (2.71, 8.35)	3.75 (2.06, 6.81)	0.000
	≥ 2 parties	99	163	10.09 (5.90,17.26)	8.16 (4.63,14.37)	0.000
Age at menarche	< 14 years	52	288	1	1	
	≥ 14years	120	336	1.82 (1.27, 2.6)	1.65 (1.13, 2.41)	0.010
Current use of family planning	Yes	115	280	2.70 (1.89, 3.83)	0.80 (0.11, 5.65)	0.82
	No	57	374	1	1	
Ever used FP	Yes	114	278	2.71 (1.9, 3.85)	1.50 (1.01, 2.23)	0.043
	No	57	376	1	1	
Dietary diversity	< 5 food groups	144	574	1	1	
	=> 5 food groups	28	80	1.39 (0.87, 2.23)	1.37 (0.82, 2.28)	0.23

## 4 Discussion

This study investigates the prevalence and factors associated with overweight/obesity among women of reproductive age in Gondar Town.

The prevalence of overweight/obesity among reproductive‐age women in Gondar Town was found to be 20.1%. This aligns with similar studies conducted in Ethiopia [25]. Kenya [26], and Bangladesh [27], which reported comparable rates. However, it surpasses findings from studies in Debre Markos [28]. Bahir Dar Town [29], and a broader Sub‐Saharan African context [1]. These disparities in prevalence could be attributed to varying lifestyle modifications, cultural dietary practices, and physical activity levels across different regions.

Conversely, the prevalence of overweight/obesity observed in this study is lower than that reported in surveys conducted in Hawasa [30], Addis Ababa [13], Ghana [31], Nepal [32], and another study in Bangladesh [33]. This discrepancy may be influenced by differences in income levels among populations.

The rate of overweight/obese rates in the study area is high, which is similar to reports from the previous study [34]. The reason might be a result of the driving of economic, technological, and societal changes.

Multivariable analysis revealed that women with menarche age greater than or equal to 14 had 1.65 times higher odds of being overweight/obese compared to those with menarche before 14 years. This finding is consistent with previous studies [35], which suggests that hormonal changes associated with ageing, such as decreased growth hormone secretion and leptin resistance, may contribute to fat accumulation.

Furthermore, the odds of being overweight/obese were 8.16 and 3.75 times higher among women with parity greater than or equal to two and parity equal to one, respectively, compared to null parity. Similar associations have been reported in studies from China [36]. Peru [37], and Iran [38]. This could be attributed to dietary habits and physical inactivity during pregnancy, leading to weight gain.

Additionally, women who had ever used family planning had 1.50 times higher odds of being overweight/obese compared to non‐users. This finding is supported by reports from India [39] and Kenya [26]. Hormonal contraception may alter nutrient metabolism and induce weight gain, while estrogen and progesterone may stimulate appetite and promote fat retention.

Obese women may be less likely to use contraception [40]. The reason is that negative attitudes and stigma surrounding obesity can create barriers to accessing reproductive health services and contraception [40]. Some studies have revealed that wealthier women are more likely to be overweight or obese [31].

Dietary patterns during and after pregnancy can significantly influence metabolic health and weight, potentially leading to overweight or obesity [41]. The reason might be that dietary patterns during pregnancy, metabolic changes postpartum, and behavioral factors significantly increase the risk of overweight and obesity in mothers.

In summary, this study underscores the importance of addressing lifestyle factors, reproductive history, and contraceptive use in efforts to combat overweight and obesity among women of reproductive age in Gondar Town.

## 5 Conclusion

In this study, overweight/obesity was high. Late age of menarche, parity, and ever‐using family planning were factors affecting overweight/obesity in reproductive‐age women. Healthcare workers should raise awareness among reproductive‐age women about the impacts of overweight/obesity. Researchers should emphasize searching for environmental risk factors, genetic predispositions, and the role of gut hormones and other factors, and implement different public health intervention strategies such as lifestyle, health services, and community support.

## 6 Strengths and Limitations

As a limitation, the investigator acknowledges there might be a social desirability bias; histories of past illnesses were assessed orally and not diagnosed by laboratory.

## 7 Policy Implications

This research finding on overweight and obesity has several policy implications, including the implementation of public health initiatives and trade policies that can address diet‐related chronic diseases.

## Author Contributions

**Mequanente Dagnaw:** conceptualization, data curation, formal analysis, methodology, software, writing review, and editing. **Melkie Mekonnen:** conceptualization, data curation, writing original draft. **Solomon Mekonnen Abebe:** supervision, data curation checkup, formal analysis checkup. **Esmeal Ali Muhammad:** supervision, data curation checkup, formal analysis checkup.

## Ethics Statement

Ethics approval was obtained from the ethical review committee of the Public Health Institute, College of Medicine and Health Sciences, University of Gondar. The research was granted clearance and approval under the reference letter Ref No/IPH 24/07/2022. The data‐gathering tool was designed to exclude any personal information, such as names or unique identifying numbers, that could be used to identify individuals.

## Consent

The aim of the study was informed to each study participant and the study participant had a right to refuse or discontinue without any restricted finally, informed oral consent was obtained from each participant before data collection, and confidentiality was assured.

## Conflicts of Interest

The authors declare no conflicts of interest.

## Transparency Statement

The lead author Mequanente Dagnaw affirms that this manuscript is an honest, accurate, and transparent account of the study being reported; that no important aspects of the study have been omitted; and that any discrepancies from the study as planned (and, if relevant, registered) have been explained.

## Data Availability

Data can be obtained upon a reasonable request. The accompanying author can provide access to the underlying data upon reasonable request. This article is freely available to the public under the Creative Commons Attribution Noncommercial (CC BY‐NC 4.0) license. This license allows others to share, modify, and distribute the article for noncommercial purposes as long as they give proper credit, indicate any changes made, and do not use it commercially. Please refer to the following link: http://creativecommons.org/licenses/by-nc/4.0/.
